# Exercising4Cognition: Can Short Bouts of Aerobic Exercise Improve Cognitive Performance in Healthy Adults for Primary Health Prevention? Previous Findings and Suggestions for the Future

**DOI:** 10.3390/healthcare13040368

**Published:** 2025-02-10

**Authors:** Cornelia Herbert

**Affiliations:** Applied Emotion and Motivation Psychology, Institute of Psychology and Education, Faculty of Engineering, Computer Science and Psychology, Ulm University, 89081 Ulm, Germany; cornelia.herbert@uni-ulm.de

**Keywords:** acute bout of aerobic exercise, cognitive performance, physical fitness, neurovisceral integration

## Abstract

**Background:** Regular physical activity (PA) and regular exercise (RE) are essential for an active and healthy lifestyle. Additionally, the short-term effects have been investigated to understand how an acute bout of exercise impacts cognitive processing, an important aspect of mental health and well-being. Previous studies have confirmed positive effects. However, several exercise factors and human factors can influence this relationship. **Aim/Methods/Results:** This perspective paper has three main objectives: firstly, discussing the exercise and human factors that influence exercise-cognition effects significantly across studies according to previous reviews and meta-analytic studies and how this influence could be explained theoretically; secondly, highlighting important knowledge gaps and research questions for future research; and thirdly, discussing what conclusion can be drawn for cognitive health promotion. A particular focus is given to the effects of acute bouts of aerobic exercise and healthy adults as an important target group for primary health prevention. **Conclusions:** The summary of previous findings shows that the effects of an acute bout of aerobic exercise on cognitive performance in healthy adults depend on (a) exercise factors such as the duration and intensity of the acute bout of exercise, (b) cognitive factors such as the type of cognitive task and domain of cognitive functions, and (c) individual factors such as the physical activity of the individuals. Still, open questions concern the ideal duration, intensity and timing of the acute bout of exercise. In particular, more research is needed to determine whether and how aerobic exercises of short duration and an intensity above and especially below moderate intensity improve cognitive functions in healthy adults. Methodologically, these factors should be addressed by multimethod designs that consider intra- and interindividual comparisons and different response levels (self-report, behavioral, psychophysiological). In conclusion, answering these questions could pave the way for recommendations on how healthcare professionals should prescribe brief aerobic exercise as a cognitive health booster in healthy young adults. To this end, concepts of extended arousal and neurovisceral integration are useful framework models to include individual factors, like self-regulatory abilities of the individual and how these influence exercise-cognition interactions and exercise motivation during, pre-to-post and across testing sessions.

## 1. Introduction

Regular physical activity (PA) and regular physical exercise (RE) are indispensable for an active and healthy lifestyle [1]. Epidemiologically, they are associated with various health benefits including a reduced risk of mortality across the lifespan [2,3]. In addition to the effects of regular physical activity and regular exercise, short-term effects have been investigated to determine how an acute bout of exercise, particularly aerobic exercise, affects cognitive processing, an important aspect of mental health and well-being. Among the various types of possible physical exercises that may affect cognitive processing, aerobic exercise has been studied very extensively in the literature. Diverse vulnerable populations and age groups have been investigated including healthy adults. During cognitive aging, adulthood is a time of cognitive achievement and peak performance [4,5]. It is therefore important to understand how cognitive processing and cognitive performance can be promoted by acute bouts of aerobics exercises, especially in healthy adults, an important target group for primary health prevention. In line with this endeavor, this perspective paper has three main objectives.

### Aim of the Present Study

The first main objective is to discuss what has been observed in previous studies in healthy adults and how the findings have been explained theoretically. The second objective is to determine which conclusions can be drawn from the evidence so far and which important knowledge gaps for future research exist concerning the specificity of exercise-cognition interactions and the conditions under which acute bouts of aerobic exercise facilitate cognitive performance in healthy adults. Thirdly, based on these two objectives, it will be discussed how answering these questions could help experts recommend acute bouts of aerobic exercise as performance boosters for enhancing cognitive health in healthy adults.

## 2. Acute Bouts of Aerobic Exercise and Cognitive Processing in Healthy Adults: Converging Findings and Influencing Factors

A literature search was conducted in line with the first objective of this perspective paper (see Aim of the Present Study Section). The search focused on previous meta-analytic studies and reviews that examined the acute effects of aerobic exercise on cognitive performance in healthy adults. Special attention was paid to meta-analytic studies and reviews that could answer which exercise-related factors and which task- and human factors influenced exercise-cognition effects in the previous studies. In addition, and where appropriate, perspective and theoretical works, collections, and individual studies were taken into account that also provided theoretical explanations of the findings. The literature research was conducted using Google Scholar or PUBMED as search engines. The following keywords were used to find the appropriate reviews and meta-analytic studies: “aerobic exercise”, “acute bout”, “single bout”, “acute aerobic exercise”, “cognition”, “cognitive processing”, “cognitive function”, “executive”, “cognitive”, “memory”, “task”, “performance”, “adults”, “healthy adults” and the logical operators “AND” or “OR” between the related terms. The keyword search yielded several hits, comprising previous and recent reviews and meta-analytic research, opinion-, perspective-, and theoretical work, and single studies, cited in the manuscript, and that provide the basis for the discussion of the following sections of this manuscript.

The results of the reviews and meta-analytic studies suggest that across studies the effects of an acute bout of aerobic exercise on cognitive processing achieve small positive effect sizes in healthy participants, e.g., [6,7,8,9,10]; for older adults, see, e.g., [11,12,13,14]. While this supports the hypothesis that acute aerobic exercise can enhance cognitive processing in healthy adults, several factors may influence these effects according to the meta-analytic research and reviews. As detailed below, exercise factors such as the intensity of the aerobic exercise (low, moderate, or intense) or the duration of the aerobic exercise, task- and cognitive factors such as the type of cognitive functions, the type of cognitive outcome variables or the time of the cognitive performance examination (e.g., before vs. after or during the acute bout of aerobic exercise, or across repeated testing sessions), as well as individual factors such as the physical or cardiovascular fitness of the participants were found to be important exercise-, cognitive- and individual factors that could influence the relationship between acute aerobic exercise and cognitive processing. These factors may determine how and under which conditions or circumstances acute aerobic exercise can positively impact cognitive processing and cognitive performance in healthy subjects. Therefore, their consideration is important for understanding the exercise-cognition effects of acute aerobic exercise and how to use them as performance boosters for cognitive health promotion in healthy adults.

### 2.1. Exercise Factors Influencing Cognitive Performance in Healthy Adults

#### 2.1.1. Exercise Intensity

According to previous reviews and meta-analytic research, cognitive performance improves best in healthy adults with aerobic exercises of moderate intensity. This seems to hold for effects examined during or after the acute bout of aerobic exercises and for aerobic exercises including but not restricted to cycling or jogging. Some reviews supported positive effects on cognitive performance for acute bouts of aerobic exercise of low intensity, e.g., [7]. In previous reviews and meta-analytic studies, acute bouts of high-intensity aerobic exercise did not achieve similar effect sizes [15]. However, previous reviews and meta-analytic research found that only a few studies used acute bouts of high-intensity exercises so far. One reason for the bias in previous studies toward choosing moderate-intensity exercise is that high-intensity aerobic exercise can reduce cognitive processing, induce cognitive load, and negatively affect mood in untrained individuals, adversely influencing cognitive performance. However, recent research [16] suggests positive effects of high-intensity interval aerobic training on certain types of cognitive processing tasks and cognitive functions. The previous reviews and meta-analyses indicated that similar to higher intensities, only a few studies included or utilized lower exercise intensities, for an overview, e.g., see [4,5,6,7,8,11].

Consequently, based on the currently available evidence, it could be suggested that acute bouts of aerobic exercise facilitate cognitive performance in healthy adults when performed at moderate exercise intensity. Although this suggestion is tempting, it is only tentative. As pointed out above, more studies are needed that allow an unbiased comparison of aerobic exercise of moderate, low, and high intensities. Furthermore, exercise intensity cannot be discussed independently from exercise duration. There is evidence that in addition to the intensity of the exercise, the effects of an acute bout of aerobic exercise on cognitive processing vary considerably with the duration of the exercise.

#### 2.1.2. Exercise Duration

Different exercise durations have been supported so far in healthy adults. The reviews and meta-analytic studies suggest mainly improvements in cognitive performance (pre-to post-exercise) when the exercise is carried out at moderate intensity and with an exercise duration of about 20 min up to 60 min [6,8]. Consequently, in healthy adults, cognitive performance should be increased after a bout of aerobic exercise of moderate intensity that is carried out for at least 20 min. However, shorter exercise durations below 20 min were also found effective, when cognitive performance was examined immediately after the acute bout of the aerobic exercise lasting for example only 11 min [7,17]. Furthermore, research that examined changes in cognitive processing during the acute bout of aerobic exercise suggests that cognitive performance can vary across the duration of the exercise [8]. Currently and considered across research, there is no conclusive evidence regarding the optimal duration of an acute bout of aerobic exercise for improving cognitive performance in healthy adults. As discussed above, the intensity and the duration of an acute bout of exercise, or a mix of both, intensity and duration, modulate cognitive performance in healthy adults. Moreover, as discussed below, the effects of both, exercise intensity and duration might have different effects on cognitive processing depending on which type of cognitive processing and cognitive performance is investigated.

### 2.2. Exercise- and Cognitive Factors

#### Exercise Intensity and Duration, Type of Cognitive Processing, and Cognitive Outcome Measures

Many different cognitive functions have been examined in previous studies. To this end, several cognitive tasks have been used including attention tasks, sensorimotor tasks, or more specifically, cognitive processing tasks that require executive functions such as cognitive inhibition, cognitive planning, or task switching. Most of the reviews and meta-analytic studies focusing on healthy adults agree that performance improvements were consistently achieved in studies that used cognitive tasks that examine executive functions, particularly tasks requiring cognitive inhibition (i.e., the ability to deliberately or unintentionally suppress the processing of task-irrelevant or distracting information). Positive effects were reported when cognitive performance was investigated after the aerobic exercise, for aerobic exercise carried out at moderate intensity and a duration of at least 20 min compared with exercises of the same intensity but with shorter or longer exercise durations [18,19,20]. In contrast, a critical time range of 15–30 min of exercise seems optimal for improving cognitive performance in memory tasks before to after an acute bout of aerobic exercise carried out at moderate intensity [20,21,22]. In addition, the same exercise duration of an aerobic exercise carried out at moderate intensity might improve cognitive performance in one type of task (e.g., memory), but have no effects or reduce the performance in other types of cognitive tasks. These effects might depend on whether cognitive performance in these tasks is investigated immediately after the exercise, for example during exercise recovery, or after longer retention intervals [20,21,23,24].

The meta-analytic and review studies that examined the effects during the exercise period likewise suggest that each type of cognitive function could benefit differently from the same acute bout of exercise. Akin to the results of pre-post exercise studies, improvements in cognitive performance have been reported preferably for tasks that require executive functions such as interference control or cognitive inhibition. In particular, positive effects on reaction time measures have been reported when exercising for approximately 20 min (including warm- and cool-down [25,26]). In contrast, opposite effects were reported when response accuracy instead of reaction time was considered as the dependent variable. Thus, differential effects of an acute bout of aerobic exercise on measures of accuracy and reaction time were observed. This was found although the aerobic exercise was of moderate intensity and the cognitive task and the exercise both lasted only a few minutes (shorter than 8 min) (e.g., [27]). Moreover, as highlighted and summarized in [9], no improvements in cognitive performance indicated by accuracy measures or reaction time measures were observed in studies that employed working memory tasks. This was found during or after the exercise for low, moderate, or vigorous aerobic exercise intensities and exercise durations above 20 min [28,29,30]. Working memory accuracy, however, was found to improve in some studies during moderate-intensity exercise of shorter durations, i.e., when the exercise lasted only a few minutes, e.g., [31]. Similar to the discussion of the role of exercise intensity, previous meta-analytic and review studies indicated a bias in previous research regarding exercise duration. As summarized above and as pointed out by previous revies and meta-analyses, e.g., [6,9,29], most studies utilized exercise durations of 20 to 60 min, while shorter durations have been used only selectively. Moreover, as discussed in the next section, none of the effects observed in previous research might apply to the average healthy adult in general.

### 2.3. Acute Bouts of Aerobic Exercise and Cognitive Performance: The Role of Individual Factors

Research has primarily concentrated on healthy adult populations, often considering them as a uniform group in terms of age and physical or mental health. Nevertheless, many previous studies included in the previous reviews or meta-analyses suggested that individual differences play a significant role. Besides age, sex, and socioeconomic variables, the cognitive and physical fitness levels of the participants have a significant impact. Positive effects were found in healthy adults with higher habitual levels of aerobic endurance and physical fitness or participants with generally lower cognitive performance [32,33,34,35,36,37].

**Figure 1:**
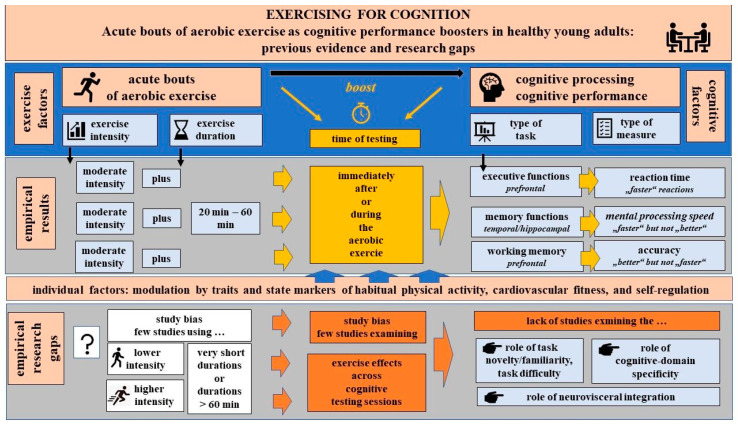
Figure 1 illustrates the current evidence discussed in Section 2 of this manuscript and the research gaps (discussed in the following sections).

Figure 1 summarizes the previous findings discussed in Section 2. The figure focuses on the effects that according to the meta-analytic research and review studies (described in Section 2) were most consistently reported. In addition, the figure highlights the limitations of previous research and research gaps that are discussed in detail in the following sections.

## 3. Explanation of the Previous Findings

### 3.1. Cognitive Domain Specificity vs. Cognitive Domain—General Effects

Despite their heterogeneity, the results of the previous research briefly summarized above are consistent with several suggestions. Firstly, the results support the hypothesis of cognitive domain-specific rather than cognitive domain-general effects. Cognitive domain specificity suggests that an acute bout of aerobic exercise can differentially affect the type of cognitive processing, its speed, and its accuracy depending on the intensity and duration of the acute bout of the aerobic exercise.

Secondly, the degree and outcome of this interaction might depend on the degree of the shared processing resources between the exercise and the cognitive task. Furthermore, the temporal relationship between the exercise and the cognitive processing plays a role, i.e., whether both tasks (exercise and cognition) are executed simultaneously or whether cognitive processing is examined within a pre-post exercise regime that furthermore considers repeated testing sessions comprising cognitive processing with and without acute aerobic exercise. Analyzing the effects across different exercise and cognitive testing sessions is of general importance and particularly important for memory tasks. Concerning memory tasks, cross-session effects are important for whether acute aerobic exercise influences learning, consolidation, forgetting, or recall when performed shortly before, after, or during these memory stages [33].

Thirdly, and in line with the suggestion of cognitive domain-specificity, the control of the tasks by different brain networks is important. In particular, cognitive processing controlled by prefrontal cortical brain networks or medial-temporal brain networks or hippocampal memory networks has been discussed in the literature [10,24,30,32,33]. Cognitive tasks controlled by these brain networks might benefit differentially from the same exercise depending on whether the task is a dual-task (competing with the exercise) or whether cognitive performance is considered in comparison from pre- to post-exercise or across testing sessions. Notably, care must be taken of which cognitive performance measures are of interest. Whether accuracy or speed of cognitive processing or both is necessary for achieving high cognitive performance might decide if performance is boosted by the acute bout of aerobic exercise pre-to post or during exercise, or across longer retention intervals.

Finally, the mental fitness and physical fitness of the individual subject constitute significant individual modulators of the cognitive effects of an acute bout of aerobic exercise. Theoretically, individual traits and states are an important source of interindividual variability (see below) that can mediate or modulate exercise‒cognition interactions among healthy adults. This can concern discussions on dose‒response relationships between the intensity and the duration of the exercise and its effects on cognitive performance [36]. Therefore, human factors require careful control in exercise studies examining acute effects of aerobic exercise on cognitive performance.

### 3.2. Theoretical Explanations of Exercise-Cognition Effects

Theoretically, several theories—physiological, biological, and cognitive—have been suggested as framework models for the exercise-cognition interactions reported above. Arousal theories (for an overview, see [38]) or the transient hypofrontality theory [39] are among the most popular theories. Arousal theories claim a U-shaped exercise-cognition relationship, whereas the transient hypofrontality theory proposes that a decrease in neural activity occurs in brain structures that are not used for performing the exercise. In addition, theories that propose interactions of arousal and hypofrontality exist. These theories suggest that exercise-induced changes in physiological arousal and prefrontal neural activity may interact differently at low, moderate, or high exercise intensities and probably vary with the exercise duration [40]. The theories suggest a linear increase in cognitive performance with increasing arousal during acute bouts of aerobic exercise of low and moderate intensities. This increase is followed by a decrease in performance due to hypofrontality, especially at higher exercise intensities or longer exercise durations. These revised arousal theories may explain several of the empirical observations outlined above. This is because interactions between exercise-induced changes in arousal and cognitive performance are also modulated by task difficulty (both exercise and cognitive task) and task novelty, e.g., for a discussion [38,40]. In this view, the effects of an acute bout of aerobic exercise on the same cognitive task can increase with increasing habit strength, as experimentally operationalized and reflected, for example, in the concept of task novelty/familiarity. This means that the effects of exercise on cognitive performance will vary not only based on the timing of the assessments (i.e., whether assessed during or after exercise) but also depending on the specific testing sessions and retention periods. In these theories, it is significant whether the cognitive tasks are performed first together with or without an aerobic exercise or later, during a second exposure together with or without an aerobic exercise.

Theories that explain why individual differences may influence the effects of aerobic exercise on cognitive processing are scarce; for a discussion, see, e.g., [41]. Models of neurovisceral integration could provide a promising theoretical framework to this end [42,43]. Neurovisceral integration models could explain the effects in healthy young adults. Adulthood is a life period characterized by ongoing cognitive development. At the neural level, this period of cognitive development in adults can be described by several changes, for an overview [43]. These changes may include changes in effective functional network connectivity among large-scale brain networks involved in cognitive control [43,44]. The theories of neurovisceral integration suggest a network of brain areas known as the ‘central autonomic network’’ (CAN) [41,45]. This network provides the cerebral interface for interactions between the cognitive, emotional, and autonomic states of an organism. The network comprises brain regions such as the anterior cingulate cortex (ACC), insula, and ventromedial prefrontal cortex. These brain regions have been shown to change their neural activity during an acute bout of aerobic exercise and to be positively correlated with the cardiorespiratory fitness level of the participants [45]. Therefore, from a developmental perspective, the CAN network can be considered an important brain network of self-regulation [41,42,45], still undergoing significant changes in young adults. Thus, by repeatedly triggering the CAN in a bottom-up manner (acute aerobic exercise acting as a stimulus that can lead to several visceral changes directly controlled by the CAN), acute bouts of aerobic exercise could improve cognitive processing via the modulation of the self-regulatory and central-autonomic capacity of the brain in terms of CAN network effectiveness. From this theoretical perspective, acute bouts of aerobic exercise could act as useful short-term boosters of cognitive performance among healthy young adults.

## 4. Research Questions for the Future

As critically discussed in this manuscript and illustrated in Figure 1, further research is required. For this purpose, key research questions can be defined based on previous studies and the theoretical models discussed above. Answering these questions will add to a still underrepresented body of literature and validate under which conditions acute bouts of aerobic exercise can boost cognitive performance in healthy adults.

In short, the key research questions for future research should be based on previous research and focus on three major aspects:

(1) Under which conditions does an acute bout of aerobic exercise modulate cognitive performance in healthy young adults?
(2) Which type of cognitive task and cognitive function including speed and accuracy of processing benefit from an acute bout of aerobic exercise?
(3) How do the effects of an acute bout of aerobic exercise on cognitive processing differ in terms of individual differences in physical and mental fitness including the self-regulatory capacities of the participants?

Table 1 provides a detailed summary of the three key aspects to guide future research.

Answering the questions summarized in Table 1 will help fill research gaps. As pointed out in this manuscript, these research gaps concern whether cognitive performance in healthy young adults improves when exercise intensity is higher than moderate and especially of lower intensity, when exercise durations are very short (e.g., less than 20 min). Cognitive performance needs to be examined in all cognitive domains across different cognitive functions, during, shortly before, and shortly after as well as during longer retention intervals after the acute bout of aerobic exercise. This will show, whether the cognitive enhancement from an acute bout of aerobic exercise is short-lived or extended across repeated sessions including cognitive testing intervals with and without exercise (see Table 1). The limitations of this perspective paper are that its conclusions focus mainly on a selection of primarily meta-analytic and review studies, which limits the methodological strength of this type of manuscript. In line with the objectives of perspective papers, the proposed research questions can guide future standardized reviews and meta-analytic studies, for a very recent approach in this direction, see [46]. They also encourage discussions about optimal study designs to effectively address these questions (see Table 1).

Research has shown that young healthy adults are a vulnerable population at risk of mental disorders and reduced well-being. According to recent surveys, several symptoms, including psychological and stress-related psychophysiological symptoms, have increased in young healthy adults, affecting their mental and physical health in both the short and long term [47,48]. This includes a large proportion of young adults of working age. Poor cognitive functions are important negative predictors of mental and physical health and psychological well-being [47,48]. In addition, psychological and mental stress can significantly impair cognitive functions [48].

Addressing the knowledge gaps discussed above will help establish recommendations for how acute bouts of exercise can serve as interventions for promoting cognitive health in adults. While international guidelines for physical activity and regular exercise to promote health for all age groups and various vulnerable target groups have been established, e.g., by the World Health Organization (WHO) and continuously developed further [10,49], there are still no comparable evidence-based recommendations or guidelines for the promotion of cognitive health of young adults by single bouts of physical activity comprising aerobic exercises.

## 5. Conclusions

For a successful transfer to practice, it must be possible to decide, based on empirical evidence, how acute bouts of aerobic exercise should be integrated into the daily work routine of young adults to increase their cognitive performance in a cognitive domain-specific manner. Next, exercise interventions consisting of brief bouts of aerobic exercise could be developed to address cognitive health promotion specifically in adults, with adulthood being the age period in which aerobic exercise can significantly enhance and protect cognitive functions [50]. Establishing guidelines for acute bouts of exercise could assist healthy individuals in combating the loss of attention, concentration, and cognitive inhibitory control associated with a primarily sedentary lifestyle, both during work and at home, for a recent approach in this direction, e.g., [51,52,53]. Ideally, these guidelines should be established by expert groups and could follow approaches as the ones suggested in [54].

Encouraging people to reduce sedentary behavior during work and at home by incorporating short physical activity breaks that involve brief sessions of aerobic exercise seems more important than ever. According to the WHO, any level of activity is better than none for promoting health from a young age. This perspective paper aimed to summarize the converging evidence about exercise-cognition effects and based on it discussed important open questions that promote research and health efforts in this direction. The manuscript revealed important open questions concerning the ideal duration, intensity and timing of the acute bout of aerobic exercise. In particular, it was shown that more research is needed to determine the circumstances under which acute bouts of aerobic exercises improve cognitive functions in healthy adults. Answering these questions could pave the way for recommendations by experts on how healthcare professionals can prescribe brief aerobic exercise as a cognitive health booster in healthy young adults. To this end, extended arousal and neurovisceral integration would be useful framework models to include individual factors, like self-regulatory abilities, and how these influence exercise-cognition interactions and exercise motivation. Knowing under which conditions, including how long and at which intensity one needs to carry out an acute bout of aerobic exercise to boost cognitive performance is an important prerequisite for enhancing exercise motivation and for motivating people to use acute bouts as physical activity breaks.

## Figures and Tables

**Table 1:** Key questions from the past to be explored in future research.

Acute Bouts of Aerobic Exercise for Cognitive Health Promotion in Young Adults Open Questions That Need To Be Addressed by Future Research	
Key Questions	Research Gaps
(RQ1) Under which conditions does an acute bout of aerobic exercise modulate cognitive performance in healthy young adults?	(a) when the exercise is carried out at different intensities, especially at low-intensity levels (b) and at a short duration (lasting only a few minutes) (c) and cognitive processing is compared across cognitive tasks and cognitive domains (see also RQ2) (d) at different points in time and not only pre- to post vs. during the exercise but across repeated sessions with vs. without exercise on the same as well as on different days (e) compared across different levels of responding (e.g., physiological, psychological, neural)
(RQ2) Which type of cognitive function benefits from the acute bout of aerobic exercise?	(a) are cognitive functions differently benefitting from an acute bout of aerobic exercise pre-post vs. during the exercise concerning the role of task novelty/familiarity the speed and accuracy of the mental processing the brain networks involved in the cognitive task and the aerobic exercise
(RQ3) How do the effects of an acute bout of exercise on cognitive performance differ in terms of individual differences?	(a) when human traits and states are considered such as the physical and mental fitness levels of the participants the degree of central autonomic self-regulation and neurovisceral integration triggered by the acute bout of aerobic exercise

## Data Availability

No new data were created or analyzed in this study.
